# Pesticides and Male Fertility: A Dangerous Crosstalk

**DOI:** 10.3390/metabo11120799

**Published:** 2021-11-25

**Authors:** Sílvia Moreira, Sara C. Pereira, Vicente Seco-Rovira, Pedro F. Oliveira, Marco G. Alves, Maria de Lourdes Pereira

**Affiliations:** 1 Department of Medical Sciences, University of Aveiro, 3810-193 Aveiro, Portugal; s.moreira@ua.pt; 2 CICECO-Aveiro Institute of Materials, University of Aveiro, 3810-193 Aveiro, Portugal; 3 QOPNA & LAQV, Department of Chemistry, University of Aveiro, 3810-193 Aveiro, Portugal; pfobox@gmail.com (P.F.O.); saracatarinapereira@gmail.com (S.C.P.); 4 Clinical and Experimental Endocrinology, Unit for Multidisciplinary Research in Biomedicine, Department of Anatomy, Institute of Biomedical Sciences Abel Salazar, University of Porto, 4050-313 Porto, Portugal; 5 Department of Cell Biology and Histology, University of Murcia, 30100 Murcia, Spain; vicente.seco@gmail.com

**Keywords:** male infertility, pesticides, EDCs, obesogens, Leydig cells, Sertoli cells, peritubular myoid cells, spermatogenesis, testicular metabolism, pesticides detection, metabolic changes detection

## Abstract

In recent decades, an increasing incidence of male infertility has been reported. Interestingly, and considering that pesticides have been used for a long time, the high incidence of this pathological state is concomitant with the increasing use of these chemicals, suggesting they are contributors for the development of human infertility. Data from literature highlight the ability of certain pesticides and/or their metabolites to persist in the environment for long periods of time, as well as to bioaccumulate in the food chain, thus contributing for their chronic exposure. Furthermore, pesticides can act as endocrine disrupting chemicals (EDCs), interfering with the normal function of natural hormones (which are responsible for the regulation of the reproductive system), or even as obesogens, promoting obesity and associated comorbidities, like infertility. Several in vitro and in vivo studies have focused on the effects and possible mechanisms of action of these pesticides on the male reproductive system that cause sundry negative effects, even though through diverse mechanisms, but all may lead to infertility. In this review, we present an up-to-date overview and discussion of the effects, and the metabolic and molecular features of pesticides on somatic cells and germinal tissues that affect germ cell differentiation.

## 1. Introduction

Infertility is a global public health problem, involving developed and developing countries [1]. As clinically defined, the term infertility is used to describe the inability of a couple to achieve pregnancy, usually after one year or more of regular and unprotected sexual intercourse [2]. Even though there are no reliable numbers for the global prevalence of infertility, recent studies have reported that infertility rates, which ranged from 7–8% in the 1960s, have increased drastically to current numbers of 20 to 35% [3]. Overall, 35% of the cases are associated with female reproductive problems, 30% are due to male reproductive problems, in 20% of cases the reason is found in men and women, and in 15% of the cases there is no identifiable cause, known as idiopathic infertility [4]. Concerning specifically male infertility, several factors can contribute to this condition such as hormonal imbalances, anatomical causes, sexually transmitted diseases, genetic factors, and environment and lifestyles [5,6]. Indeed, among the idiopathic risk factors is found environmental or occupational exposure to toxicants, like pesticides [7].

The use of pesticides is almost as old as agriculture. People, such those in China, Greece and Sumer, before Christ, had already used the ability of sulfur powder to control insects and salt in killing weeds. Over time, they also noticed that certain plants functioned perfectly as a potent poison for most vertebrates and invertebrates. However, the “official” use of pesticides only began in the late 19th century, with the sale of some inorganic salts. Subsequently, most of these salts were considered toxic to pests and humans and, therefore, replaced by organic compounds [8]. Thus, pesticides can be defined as chemicals, natural or synthetic, used in various agricultural practices to control pests, weeds and plant diseases [9], in order to enhance food production, and help production processing, storage, transport or marketing of the food and agriculture products [10]. Although the use of these chemicals has been of great benefit to human life, increasing evidence has demonstrated several negative effects on the environment and human health. In fact, exposure to pesticides has been associated with the incidence of different chronic diseases such as cancer, birth defects and developmental toxicity, reproductive disorders, diabetes, cardiovascular diseases, central nervous system pathologies, like Parkinson, Alzheimer or Amyotrophic Lateral Sclerosis (ALS), amongst other chronic diseases [11].

Pesticides can act as endocrine disrupting chemicals (EDCs), able to interfere in the synthesis, secretion, transport, binding, action or elimination of natural hormones, responsible for the maintenance of homeostasis of events such as reproduction and development [12]. Additionally, certain pesticides can, also, promote obesity acting as obesogens [13]. This term refers to compounds present in the environment and/or diet that actively stimulate adipogenesis and promote associated comorbidities, among which is infertility [14]. As a matter of fact, since the 1970s, obesity has tripled [15], and, nowadays, is recognized that accumulation of toxic substances and lipid-soluble endocrine disruptors in fat tissue contributes to the amplification of the deleterious effects induced by increased body weight [16]. In line with this fact, storage of toxicants and obesogenic compounds in reproductive organs has been described, due to the high lipid content of these tissues, which may cause testicular dysgenesis syndrome, including atrophy of seminiferous tubules and germ cell degeneration [17]. Besides, when there is excessive fat tissue, more testosterone is converted to estrogen, thus reducing testosterone levels and increasing estradiol ones. As such, it is rightful to infer that obesity promotes hormone dysregulation [18]. Indeed, the hypothalamus-pituitary-testicular (HPT) axis, as well as the signals coming from this complex system, are responsible for the adequate male reproductive function, including its metabolism, and, therefore, in an obesity condition, infertility can be enhanced [19]. Since the growing infertility and obesity conditions started around the same period of time, and both seem to be associated with exposure to pesticides, at least to some extent, it is plausible to assume that, in truth, pesticides are the principal cause behind this increasing disease cases.

In this review, we focus on the effects and mechanisms of action of pesticides with potential obesogenic action on the somatic testicular cells, testicular tissue, and metabolism that may result in male infertility. We also aimed to highlight the most common routes of exposure to pesticides, and to explain some aspects concerning each class of pesticides, in order to better understand how they can enter the human body, and affect specifically the male reproductive system. Finally, some methodologies used for the detection of pesticide and their metabolites, as well as metabolic pathway changes will be discussed.

## 2. Routes of Exposure to Pesticides

Pesticides are mainly used in agricultural practice, to increase crop productivity, but other applications include home gardens, handling with domestic animals by the use of dewormers, or even to control infectious diseases. So, human exposure to pesticides can occur through many routes, like occupational dealing with production, transport, delivery and application of pesticides themselves, residing in places with high incidence of pesticide residues, as well as circulation and accumulation of these chemicals in the food chain [20].

Depending on the amount of pesticides used, they can accumulate in plants, water, soil, air and biota, meaning they can contaminate soil and water, remain in crops, and finally enter the food chain, thereby posing a threat to humans [9]. Indeed, fruits and vegetables, which are strongly encouraged to be consumed given their richness in essential nutrients, not only to prevent vitamin deficiency, but also to reduce the incidence of certain pathologies, such as cancer, cardiovascular diseases, obesity, among others, are often the top of contaminated food [21]. In this way, diet is the main source of exposure to pesticides, the so-called chronic exposure, for the majority of the worldwide population, and a concerning fact resides in the limited knowledge on the chronic adverse effects of low doses, compared to the knowledge on the acute toxic effects observed in humans, upon suden exposure to high doses of pesticides [22,23]. Moreover, the presence of pesticide residues and/or their degradation products can even be more toxic than their precursors [24]. Nonetheless, and even though there are alternatives to avoid the use of chemical pesticides, such as application of various biopesticides or the development of some pest-resistant crop varieties, using transgenic approaches, traditional pesticides are still preferred over all other options to protect crops from yield loss [9].

## 3. Classes of Pesticides—A Brief Description

Until the late 1940s, natural and inorganic pesticides, like lead arsenate, were the only pesticides used, until their replacement by new and potent developed synthetic organic chemicals [8]. Chronologically, the first to be introduced were the organochlorine pesticides (OCPs), followed in by the organophosphates (OPs), carbamates, pyrethroids, phenylpyrazoles, and more recently, the neonicotinoids (Table 1) [25].

## 4. Effects and Mechanism of Action of Pesticides on Testicular Somatic Cells

Spermatogenesis requires the precise regulation of the somatic cell populations, Leydig, Sertoli and peritubular myoid cells [29]. Altogether with the germ cells, they represent the major testicular cell types [30]. Despite the different characteristics and functions of each cell type, they are all susceptible to internal and external factors, such as, environmental factors, hormonal deregulation, diseases and oxidative stress [30,31,32]. Considering specifically pesticides, they can damage the male reproductive system in several ways: (i) reproductive toxicity with direct damage to cell’s structure; (ii) changes in DNA structure, leading to gene mutations that may cause birth defects or inability to conceive; (iii) epigenetic effects induced by changing the way genes are expressed. In fact, there are evidences that associated with exposure to environmental factors are modifications in the genome. In this way, disorders may be inherited from father to child, by epigenetic components of the cell, like DNA methylation, histone modification and non-coding RNAs [33]. Moreover, pesticides can damage the male reproductive system by acting as EDCs. Indeed, the vast majority of pesticides are classified as EDCs [34]. Additionally, and as mentioned before, pesticides can act as obesogens, being 30 to 40% of all cases of male infertility closely associated with obesity [14,35].

### 4.1. Leydig Cells

Leydig cells are responsible for the synthesis of testosterone, essential for spermatogenesis, sperm maturation, and sexual function in adults, and for the masculinization of the male foetus in utero [31].

**Figure 1:**
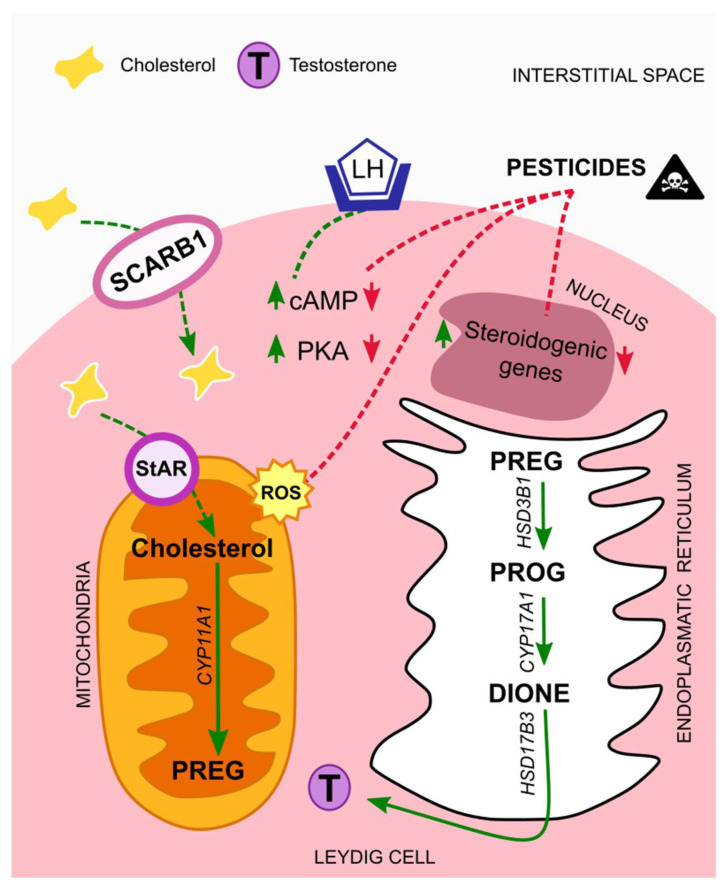
Mechanisms of action of pesticides on Leydig cells. In normal situations the luteinizing hormone (LH) binds to its receptor, LH/choriogonadotropin (LHCGR), present in Leydig cells membrane´s. The levels of cAMP and PKA increase, thus initiating a cascade that leads to testosterone synthesis. Exposure to pesticides interfere with testosterone synthesis cascade, by down-regulating cAMP and PKA levels, and by suppressing the expression of steroidogenic genes. Moreover, it leads to elevated amounts of ROS that contribute to mitochondrial damages. Abbreviations correspond to: LH—luteinizing hormone; SCARB1—scavenger receptor class B type I; StAR—steroidogenic acute regulatory protein; cAMP—cyclic adenosine monophosphate; PKA—protein kinase A; PREG—pregnenolone; PROG—progesterone; DIONE—androstenedione; T—testosterone; ROS—reactive oxygen species.

Biosynthesis of testosterone follows a cascade that starts with cholesterol as a substrate. This lipid has different sources; it can be synthesized de novo from acetate and stored in lipid droplets, or it can derive from Leydig cell membranes, like the plasma membrane. In either case, cholesterol is transferred from the outer to the inner mitochondrial membrane through the steroidogenic acute regulatory protein (StAR), known as the rate-limiting step [36]. On the other hand, it can be transported from extracellular high-density lipoprotein via its receptor, SCARB1. The expression of both StAR and SCARB1 is regulated by the luteinizing hormone (LH), via the LH/choriogonadotropin receptor (LHCGR). In more detail, LH binds to the LHCGR, thus triggering adenylate cyclase activity, which increases intracellular cAMP levels and cAMP-dependent phosphorylation of proteins through protein kinase A (PKA). In this way, both trophic and acute LH effects are allowed to initiate [31,37]. After LH regulation and before testosterone formation, cholesterol must be cleaved by the cytochrome P450 cholesterol side chain cleavage enzyme (CYP11A1), originating pregnenolone (PREG). Next, PREG is cleaved by the 3β-hydroxysteroid dehydrogenase isoform 1 (HSD3B1), giving rise to progesterone (PROG) which is then cleaved by the cytochrome P450 17a-hydroxylase/17,20-lysase (CYP17A1), forming androstenedione (DIONE). Finally, testosterone is formed by DIONE cleavage through the 17b-hydroxysteroid dehydrogenase isoform 3 (HSD17B3) (Figure 1) [38].

In a recent study performed by Dong et al. (2020), male rats and isolated Leydig cells were exposed to dimethoate, an organophosphate insecticide, proposed to act as an endocrine disruptor. The results obtained showed diminished serum levels of testosterone, while serum LH and follicle-stimulating hormone (FSH) levels increased, something that may be explained by the normal negative feedback from the HPT axis [5]. Moreover, the authors observed a reduction in the size of Leydig cells, as well as down-regulation of the genes *Star*, *Cyp11a1* and *Hsd3b1* and their protein expression [39]. These results may be complemented on the bases of another study. Vega et al. (2015) demonstrated that *Nr0b2* expression, which is regulated by the HP axis and has been proposed to contribute to local regulation of steroidogenesis [40], it is also involved in mediating the deleterious effects of EDCs. Indeed, in the presence of those chemicals, there is the transformation of cAMP into AMP, thus activating AMPK, which represses steroidogenesis. AMPK activation allows the expression of *Nr0b2*, while the expression of steroidogenic genes remains low, and so the levels of steroid hormones [41]. In line with these findings, a study with chlorpyrifos, an organophosphorus pesticide, demonstrated that in the presence of this compound, AMPK was activated, inducing reproductive toxicity [42]. Interestingly, Eze et al. (2019) showed that exposure of a Leydig cell line (MA-10) to p,p′-DDT, known as a persistent organochloride pesticide, produced a biphasic effect. In low doses, p,p′-DDT increased the levels of testosterone, whilst in high doses the opposite was observed. Surprisingly, the levels of PROG increased in a dose dependent manner. As explained by authors, this biphasic effect can occur through three distinct routes: (i) activation of G protein-coupled oestrogen receptor (GPER), instead of the classical nuclear estrogenic receptors (ER), (ii) dysregulation of the activity of the enzymes involved in steroidogenesis, and (iii) disruption of the gene expression of molecules that regulate lipid homeostasis and steroidogenesis. In fact, and as also explained, p,p′-DDT has been proved to bind and/or activate GPER, leading to stimulation of cAMP production, consequently stimulating steroidogenesis [43], which may be the case in exposure to low doses. Previously, Liu et al. (2018) had already demonstrated the capacity of ziram, considered as an endocrine disruptor, to produce a biphasic effect on the number of fetal Leydig cells [44]. In another study [45], it was demonstrated that testosterone levels diminished after rat or Leydig cells exposure to acetamiprid, a neonicotinoid insecticide. In this case, the authors observed fewer Leydig cells, after acetamiprid exposure, which may explain the reduced levels of T biosynthesis. On the other hand, it was also observed that the production of ATP and cAMP decreased, as well as the levels of enzymes involved in the biosynthesis of testosterone, which can also explain testosterone decreased levels. Moreover, an abnormal increase in the levels of reactive oxygen species (ROS) occurred, which has been demonstrated to cause mitochondrial damage, and so leading to reduced levels of ATP, that is synthesized in the mitochondria. Consequently, the levels of cAMP will also be reduced, since this second messenger is synthesized from ATP, and because it is responsible for the stimulation of the expression of genes encoding steroidogenic enzymes, this help to explain the reduced levels of those same enzymes [45].

In sum, from all the above-mentioned studies a common effect of all classes of pesticides on Leydig cells is the reduction of testosterone levels, and two main mechanism seem to be behind it. First, the endocrine disrupting properties of the compounds that leads to the suppression of genes involved in the steroidogenic process, and a second one, associated with mitochondrial damages, caused by elevated levels of ROS, which are induced by the presence of these compounds in the organisms (Figure 1).

### 4.2. Peritubular Myoid Cells

The peritubular myoid cells are the main cellular component of the seminiferous tubules wall, and are thought to play a crucial role for the intratesticular transport of immotile sperm. Furthermore, together with Sertoli cells, they form the basal lamina in the seminiferous tubule, providing the niche for spermatogonial stem cells to self-renew and maintain the pool of stem cells in the testis, as well as to pursue to differentiate into spermatocytes and sperm [29]. Despite its importance, studies on the effects and possible mechanisms of action of pesticides on these particular cell types are very scarce.

Silvestroni et al. (1999) tested the organochlorine insecticide, lindane, on the smooth muscular component of the rat testis, and observed that this chemical can change membrane polarity, as well as interfere in the rearrengment of lipids in the membranes. Besides, it was observed an increase in intracellular calcium concentration, which helped to alter the membrane potential of peritubular myoid cells that became depolarized [46]. A couple of years later, in another study with other organochlorine insecticide, endosulfan, it was demonstrated that after exposure to this compound, a significant reduction occurred in the percentage of perimeter length occupied by desmin positive cells, being desmin expressed by peritubular cells [47]. Altogether, these results shed light on the effects and possible mechanism of action of pesticides on peritubular myoid cells. However, more studies are needed to better elucidate the effects and mechanism of action of currently used pesticides.

### 4.3. Sertoli Cells

Sertoli cells are responsible for the nutritional support and a source of energy to the developing germ cells. They produce lactate, via the metabolism of glucose, preferentially, which will then be used by the germ cells to produce ATP. In addition, Sertoli cells generate and maintain the blood-testis barrier (BTB), and so, if this barrier is dysfunctional, germ cell differentiation is arrested [32].

Recently, a study with methyl parathion, an organophosphorus pesticide, showed BTB integrity alterations, as well as low sperm quality, caused by oxidative stress. The authors coadministred methyl parathion with α-tocopherol, an antioxidant, and observed no biotin infiltration in the adluminal compartment of the seminiferous tubules, whereas in the absence of this antioxidant, permeability of the BTB was observed. The BTB permeability is regulated, among other factors, by tight, adherent and gap junctions present in Sertoli cells, thus indicating a possible toxic mechanism of methyl parathion through impairments in these cells and/or their junctions [48]. In accordance with the previous study, Pisani et al. (2016) demonstrated stiffeness of the BTB junctions after exposure to carbendazim, a carbamate fungicide, consequently affecting germ cell migration [49]. Furthermore, the biochemical characteristics of pesticides can help them to cross the BTB and induce cell death. As reported by de Carvalho et al. (2020), short- and long-term exposure of Sertoli cells to methamidophos, an organophosphate, showed a reduced number of these cells, which was probably caused by direct damage, since the compound has both hydrophilic and hydrophobic domains, and thus can pass the BTB by diffusion [50]. Moreover, Rastogi et al. (2014), demonstrated that endosulfan, a chlorinated pesticide, can cause apoptosis in rat Sertoli cells by mitochondria mediated intrinsic cell death pathway, due to a significant increase in ROS and malondialdehyde (MDA) that increase lipid peroxidation, thus changing cell membrane integrity [51]. In corroboration of the previous study, Hung et al. (2015) showed that exposure of testicular cells to terbufos, an organophosphorus insecticide and nematicide, also caused an elevated production of ROS, thus reducing mitochondria membrane potential and increasing DNA damage, leading to apoptosis [52]. In addition, Ham et al. (2020) demonstrated impairments on Sertoli cell function, upon exposure to bifenthrin, a pyrethroid insecticide that correlated with mitochondrial dysfunction. However, in this study, the mitochondrial dysfunctions were associated with a dysregulation in calcium homeostasis and endoplasmic reticulum stress [53]. On the other hand, exposure of Sertoli cells to p,p′-DDE, a metabolite of dichlorodiphenyltrichloroethane (DDT), resulted in decreased FSH, and mRNA and protein expression levels of vimentin and N-cadherin. As explained, FSH participate in Sertoli cells maturation, regulate the production of specific proteins by Sertoli cells, and maintain a normal spermatogensis, besides from affecting the expression of vimentin and N-cadherin. In this way, and as proposed by the authors, toxic interference in the FSH receptor, which is exclusive of Sertoli cells within the testicular environment, may be another potential mechanism of dysregulation of these cells [54]. Moreover, a study with cypermethrin, a synthetic pyrethroid pesticide, showed decreased viability and proliferation of mouse Sertoli cells, as well as disturbances on their normal function, caused by the anti-androgenic activities of cypermethrin. In more detail, this compound was able to inhibit the activation of signaling molecules, the AR-Src interaction, and the CREB-regulated gene expression in testosterone-mediated MAPK signalling pathway, meaning that cypermethrin toxic effects on Sertoli cells was mediated by non-classical testosterone signalling pathway activation of MAPK cascade [55]. Additionally, in another study, the levels of androgen receptor (AR) were evaluated, after exposure to fipronil, a phenylpyrazole insecticide, and no changes were observed whether in AR levels, or Sertoli cell and sperm count, which depend on the expression of AR. Notwithstanding, it was observed a reduction in sperm motility, indicating that this compound may also exert his toxic effects on the epididymis, where sperm maturation occurs [56].

**Figure 2:**
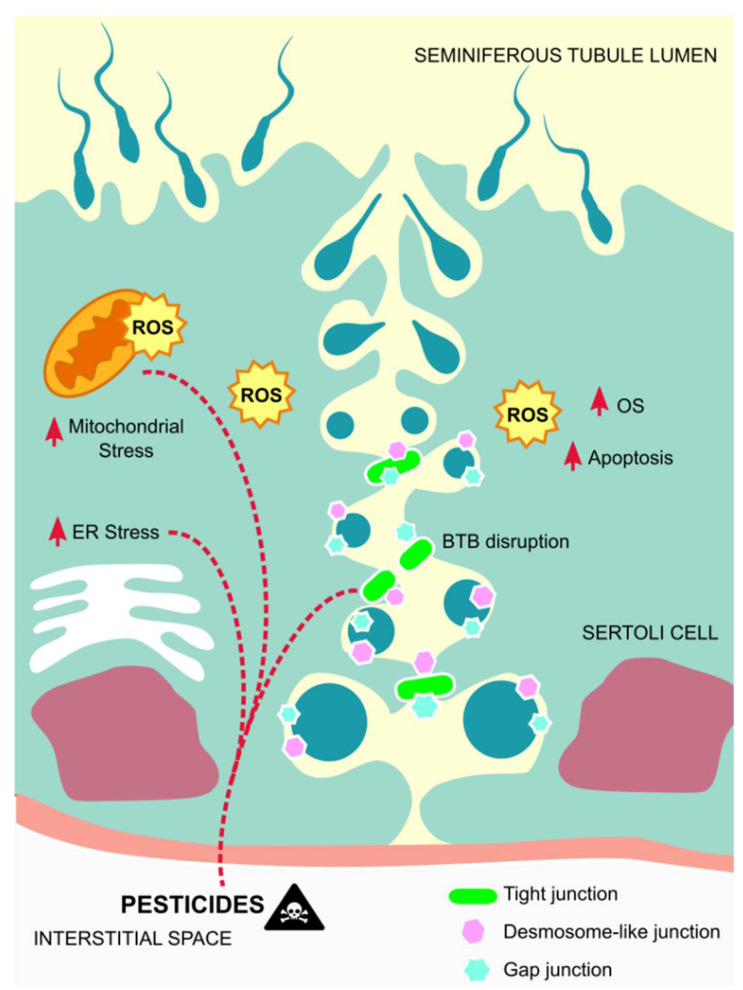
Mechanisms of action of pesticides on Sertoli cells. Exposure to pesticides leads to elevated amounts of ROS that contribute to mitochondrial damages and endoplasmic reticulum stress, as well as to Sertoli cells´ apoptosis, thus disrupting the BTB and inducing germ cells´ apoptosis. Abbreviations correspond to: ROS—reactive oxygen species; OS—oxidative stress; ER—endoplasmic reticulum; BTB—blood-testis-barrier.

In general, the effects of pesticides in Sertoli cells correlate with the apoptosis of these cells, which in most cases is associated with mitochondrial damages caused by oxidative stress, or direct induction of dysfunctions in these cells, thus not permiting them to perform their functions correctly (Figure 2).

## 5. Effects and Mechanisms of Action of Pesticides on Testicular Tissue

The testis are crucial for the synthesis of steroid hormones (steroidogenesis), and the production of mature sperm (spermatogenesis), two major functions that are only possible due to the coordination of their various cell types, as mentioned above [57]. Furthermore, Sertoli cells are responsible for forming the internal epithelium of the seminiferous tubules, while the peritubular cells, also known as smooth muscle-like cells, form the external layers. Between adjacent Sertoli cells, the network of tight junctions forms the BTB, and compartmentalize the seminiferous tubules in basal and adluminal compartments that are supported by the interstitial tissue, which supplies oxygen, nutrients as well as paracrine and endocrine hormones [58].

A study with endosulfan demonstrated its maximum effect on testes, leading to changes on this organ, as well as depletion of testicular-cell populations and sperm count and motility. Interestingly, male rats exposed to this compound were more severely affected than the female rats, something that may be associated with the specificity of endosulfan to act on the AR that is more abundant in males [59]. More recently, Erthal et al. (2020) showed that rats exposed to malathion, an organophosphate insecticide, suffered spermatogenesis and sperm release retardation, which agrees, first with a reduction, and then an increase of the seminiferous tubules, being closely associated with an alteration in spermatogenesis kinetics. As the authors explain, the mechanism behind these alterations may be associated with oxidative stress [60]. In a study performed by Elsharkawy et al. (2014), with rats exposed to chlorpyrifos, an organophosphorus compound, a significant decrease in the number of spermatogenic cells was observed in the seminiferous tubules and in the mean number of different germ cell types in all stages, as well as necrosis in some tubules along with edema in the interstitial tissue. These results are in agreement with the decrease observed in the testicular antioxidant system. Besides, the mean number and nuclear volume of the Leydig cells were also significantly decreased compared to control, consequently affecting the levels of testosterone, and indicating a possible endocrine disrupting role of this chemical [61]. Consistent with the previous study, also fipronil, which belongs to the phenylpyrazole family, demonstrated to cause histopathological alterations in the seminiferous tubules of rats. These observations were the result of oxidative damage, due to an increase in MDA and nitric oxide (NO) levels, and a decrease in the activity of antioxidant enzymes [62]. Moreover, Mohamed et al., (2019) corroborated the negative impact of oxidative stress on testicular tissue, this time by exposing rats to fenpropathrin, a member of the synthetic pyrethroids. Concurrent with the results of previous studies, the authors demonstrated that the observed germ cell apoptosis occurred through the intrinsic pathway, also known as the mitochondrial pathway, as a result of the damage caused in this organelle by the oxidative stress [63]. In addition, also imidacloprid, a member of the neonicotinoid family, whether alone or in conjugation with arsenic, was demonstrated to cause severe degenerative changes in seminiferous tubules, shrinkage, decreased lumen diameter with generalized necrosis, and depletion of germ cells. Even though the mechanism underlying these effects was not explored, once again, an increased generation of free radicals was observed, which may imply oxidative stress as the cause for these histopathological damage [64]. In agreement with this hypothesis, a study performed with mancozeb, a carbamate fungicide, demonstrated that along with apoptosis, histopathological changes, decreased sperm count in the epididymis, and elevated levels of oxidative stress in the testis were found [65].

**Figure 3:**
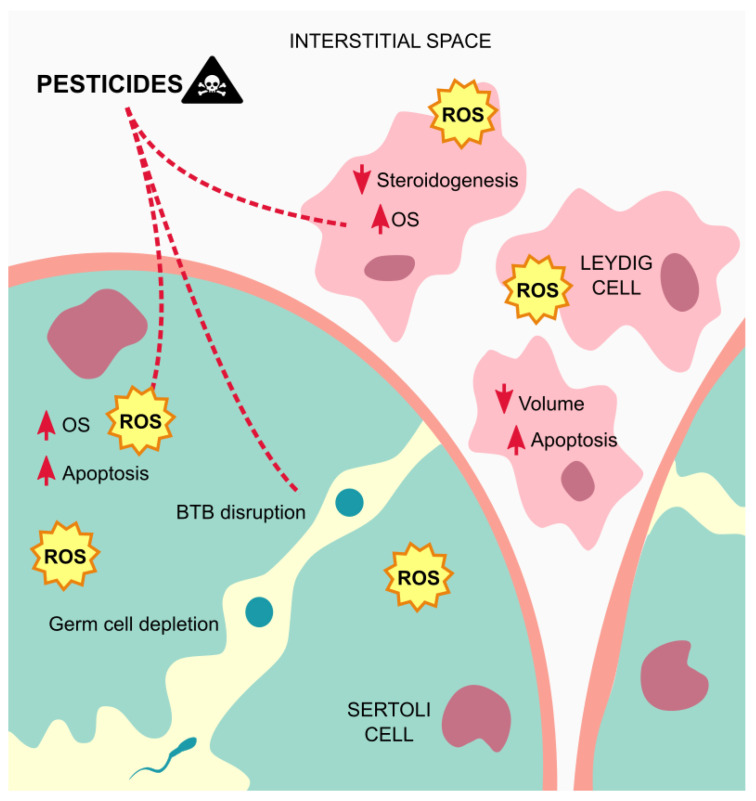
Mechanisms of action of pesticides on the testicular tissue. Exposure to pesticides leads to elevated amounts of ROS that contribute to testicular cells dysfunctions and apoptosis, and germ cells depletion, besides from inducing edema on the tissue. Abbreviations correspond to: OS—oxidative stress; ROS—reactive oxygen species; BTB—blood-testis barrier.

Overall, the seminiferous tubules seem to be the more affected testicular structure by exposure to pesticides, which often induces necrosis and edema in this tissue, due to oxidative stress (Figure 3).

## 6. Effects and Mechanisms of Action of Pesticides on Testicular Metabolism

As mentioned before, Sertoli cells establish an intimate metabolic cooperation with germ cells, which is vital for spermatogenesis. Germ cells use lactate as a substrate for ATP production [32]. This metabolite is derived, especially, from the anaerobic lactic fermentation pathway, and involves a series of steps, such as glucose uptake by GLUT1 and GLUT3, conversion to pyruvate, subsequent transformation to lactate catalyzed by LDH, and, finally, its export to germ cells through the MCT4 present in Sertoli cells [66]. In this way, when it comes to testicular metabolism, it is fundamental to underline the role of Sertoli cells [67]. As described, Sertoli cells have different roles in germ cell development that goes from physical support and immunoprotection, to the supply of nutrients and other factors. A number of different hormones and EDCs are known to be metabolic modulators of Sertoli cells [32]. For instance, the effect of the obesogen tributyltin (TBT) on the metabolism of Sertoli cells was studied, which showed a modulation of the expression and function of key intervenients in the glycolytic flux, compromising Sertoli cells metabolism. Namely, TBT 10 nM induced a significant decrease in glucose and pyruvate consumption, and in the expression of PFK1 that is responsible for the irreversible conversion of fructose-6-phosphate to fructose-1-6-bis-phosphate, in a rate-limiting step, as well as production of lower amounts of alanine, suggesting a switch in metabolism from alanine towards pyruvate. Interestingly, at 0.1 nM TBT caused more striking changes in the expression of key players in the glycolytic pathway, which indicates that tributyltin is even more effective at lower doses [68]. This observation is in accordance with a statement from Sengupta and Banerjee (2013), where the authors explain that EDCs do not follow the classic dose-response effect, and so low doses may result in stronger effects than high doses [69]. In another study, it was demonstrated that 2,4-dichlorophenoxyacetic acid (2,4-D) induced several alterarions on Sertoli cells metabolism, when these cells were exposed to concentrations from 10 µM to 10 mM. At 10 µM, a significant decrease in SCs intracellular lactate and lactate/alanine ratio was observed. In this way, and since lactate/alanine ratio is related to the NADH/NAD^+^ ratio and is intimately linked the redox state of the cell, it was possible to conclude that this compound, at the aforementioned concentration, induced a reduced cytosolic state. At 10 mM, a switch from lactate metabolism to the Krebs cycle was observed, which is not Sertoli cells preferred metabolic pathway [70]. On the other hand, 2,4-D was also found to decrease cholesterol contents in testosterone-synthesizing Leydig cells, due to suppression of mRNAs of rate-limiting enzymes in de novo cholesterol-synthesizing pathway, along with decreases in testicular testosterone synthesis. These alterations were associated with spermatocyte/Sertoli cell damages. Moreover, since these abnormalities were not detected in Ppara-null mice, it is possible to speculate that 2,4-D treatment disrupts cholesterol/testosterone homeostasis in Leydig cells through peroxisome proliferator-activated receptor alpha (PPARα) [71]. PPARα together with PPARβ/δ and PPARγ are ligand-activated transcription factors, members of the nuclear-hormone receptor superfamily [72]. Their function is to control metabolic pathways involved in lipid and energy metabolism, by sensing fatty acids and fatty acids derivatives [73]. Indeed, it was shown that PPARα and PPARβ/δ activation could stimulate the expression of genes involved in fatty acids transport and oxidation in Sertoli cells [74]. Furthermore, exposure to both chlorpyrifos, an organophosphate compound, and ethylene thiourea, the main metabolite of mancozeb, showed to interfere with thyroid hormones that are essential regulators of testis functionality, by reducing testicular T3-metabolism signaling in the exposed mice [75]. In essence, these pesticides can interfere in testicular metabolism through multiple mechanisms. It can be due to direct modulation of expression and function of metabolic pathways, through receptors involved in energy metabolism, or even by interference with hormones responsible for the normal male reproduction.

## 7. How to Detect Pesticides and Their Impact on Cellular Metabolism for Male Fertility Assessment?

Routine methods used to identify pesticides and/or their metabolites, for the evaluation of male fertility include semen analysis, measurement of reproductive hormones in the blood, and analysis of urine samples. Compared to serum analysis, the results of multiple studies are sometimes considered confusing, with seminal fluid and sperm considered not ideal samples. Higher concentrations of pesticides are consistently described in blood and urine compared to those of seminal fluid. Notwithstanding, urine analysis are also considered inadequate to illustrate the long-term effects of exposure to pesticides [10]. Due to the variety of pesticides and trace detection requirements, high precision chromatographic methods with detection by mass spectrometry are needed for urine samples. However, these methods are very expensive, and the laboratories certified for urinalysis of pesticides are limited, leading to long waiting times. In this way, other techniques, like vibrational spectroscopy are also suitable for pesticide identification. For instance, surface-enhanced Raman spectroscopy (SERS) is sensitive enough to detect pesticides in the part per trillion (ppt) range. In SERS, the laser examines an area of a microscopically roughened or “activated” gold or silver surface that has an attached surface plasmon. Thus, the vibrational modes of adsorbed analyte within the examined volume, which are involved with the surface interaction, are enhanced by factors of up to 10^9^, enabling trace detection [76].

Recently, a method to assess how pesticides and their metabolites affect male fertility and interfere with cellular metabolism has been described. For this purpose, Dudek and colleagues developed a mass isotopolome analysis for mode of action identification (MIAMI) that can detect, analyze and visualize changes in global metabolic flux. This software takes into account all known and unknown detectable metabolites in gas chromatography-mass spectrometry (GC/MS) datasets and detects changes in mass isotopomer distributions (MIDs) between conditions [77]. Previously, another software that requires no a priori information on the biological system was described, which was the non-targeted tracer fate detection (NTFD), which is able to detect all observable metabolites labeled by a stable isotope tracer within a GC/MS dataset. Moreover, NTFD calculates MIDs for all ions derived from labeled compounds, which are corrected for natural isotope abundance. Since changes in intracellular reaction rates are directly reflected in the MIDs of metabolic intermediates, it is possible to study the metabolic fluxes and enzyme activities [78].

## 8. Materials and Methods

The scientific papers mentioned and discussed in this review were collected by searching the database PubMed. Preference was given to articles published on the last five years, even though some had to be from a longer period due to the historical view, as well as the fact that for some keywords there were not to many studies published recently. To identify relevant papers the following keywords were combined: “pesticides/obesogens AND male infertility”; “organochlorine pesticides AND Leydig cells/peritubular myoid cells/Sertoli cells/testicular tissue”; “organophosphate pesticides AND Leydig cells/peritubular myoid cells/Sertoli cells/testicular tissue”; “carbamates AND Leydig cells/peritubular myoid cells/Sertoli cells/testicular tissue”; “pyrethroids AND Leydig cells/peritubular myoid cells/Sertoli cells/testicular tissue”; “phenylpyrazoles AND Leydig cells/peritubular myoid cells/Sertoli cells/testicular tissue”; “neonicotinoids AND Leydig cells”; “neonicotinoids/peritubular myoid cells/Sertoli cells/testicular tissue”; “pesticides AND testicular metabolism”; “ppar alpha AND testes”; “pesticide metabolites detection” and “metabolic pathway changes detection”. Only papers written in English were considered in this review. Relevant articles, at least to some degree, had to examine the relationship between pesticides and impaired male fertility, how to detect metabolites and metabolic pathway changes, and to give details on pesticides and male infertility.

## 9. Conclusions

Although the use of pesticides has been done for a long time, only since the beginning of their increasing use, almost concomitantly, a decrease in male fertility and an increase in obesity have been reported. Thus, despite the broad spectrum of possibilities pointed out for the development of male infertility, exposure to pesticides, particularly chronic exposure, should be considered as a preponderant factor. As discussed through this review, several negative effects on the male reproductive system come from the exposure to these chemicals, with endocrine disruption and obesogenic properties, which may lead to infertility. Despite their different characteristics, some classes of pesticides produce the same effects and/or act by the same mechanisms to induce damage at different levels in the testicular cells, tissue, and metabolism. Nonetheless, there is still a long way to go, until their effects and cellular and molecular pathways in the male reproductive system are fully characterized.

## Figures and Tables

**Table 1:** Summary of the properties and mechanisms of action of each class of pesticides.

Classes of Pesticides	Properties	Mechanisms of Action	Examples
Organochlorine pesticides (OCPs) [25]	Chlorinated hydrocarbon compounds	Alter ion exchange dynamics in axons, in the peripheral and central nervous systems, leading to decreased action potentials	Dichlorodiphenyltrichloroethane (DDT), Hexachlorobenzene (HCB)
Organophosphates (OPs) [26]	Esters of phosphoric acid	Induce irreversible inhibition of acetylcholinesterase enzyme (AChE), causing accumulation of acetylcholine (ACh) in muscarinic and nicotinic cholinergic synapses, consequently, overstimulating ACh receptors in the nervous system and neuromuscular junctions	Malathion, Parathion, Diazinon, Dichlorvos, Chlorpyrifos, Tribufos (DEF)
Carbamates [27]	Esters of N-methyl carbamic acid	Reversibly inhibit the AChE, which catalyzes the hydrolysis of ACh, leading to its increase in nerve synapses and neuromuscular junctions, triggering increased stimulation of these nerve endings	Carbendazim, Carbaryl, Aminocarb, Thiodicarb, Carbofuran, Mancozeb
Pyrethroids [25]	Synthetic pesticides based on the chemistry of natural pyrethrins	Prevent the closure of voltage-gated sodium channels in axonal membranes, blocking normal nerve impulses, thereby paralyzing and, eventually, killing the organism	Cypermethrin, Bifenthrin, Fenvalerate, Permethrin
Phenylpyrazoles [28]	Chemical structure characterized by a central pyrazole ring with a phenyl group attached to one of the nitrogen atoms of the pyrazole	Block non-competitive gamma-aminobutyric acid (GABA)-gated chloride channels, creating excessive neuronal stimulation and death	Fipronil, Pyriprole
Neonicotinoids [28]	Structurally similar to the natural insecticide nicotine	Enhanced selectivity and potency to bind to nicotinic ACh receptors (nAChRs), leading to a large influx of cations into the postsynaptic membrane of nerve cells in the central nervous system, triggering excessive excitatory neurotransmission, which results in paralysis and death	Imidacloprid, Acetamiprid, Chlothianidin
